# An Ensemble Prognostic Model for Colorectal Cancer

**DOI:** 10.1371/journal.pone.0063494

**Published:** 2013-05-02

**Authors:** Bi-Qing Li, Tao Huang, Jian Zhang, Ning Zhang, Guo-Hua Huang, Lei Liu, Yu-Dong Cai

**Affiliations:** 1 Institute of Systems Biology, Shanghai University, Shanghai, P. R. China; 2 Key Laboratory of Systems Biology, Shanghai Institutes for Biological Sciences, Chinese Academy of Sciences, Shanghai, P. R. China; 3 Department of Genetics and Genomic Sciences, Mount Sinai School of Medicine, New York City, New York, United States of America; 4 Department of Ophthalmology, Shanghai First People's Hospital Affiliated to Shanghai Jiaotong University, Shanghai, P. R. China; 5 Department of Biomedical Engineering, Tianjin University, Tianjin Key Lab of BME Measurement, Tianjin, P. R. China; University of Akron, United States of America

## Abstract

Colorectal cancer can be grouped into Dukes A, B, C, and D stages based on its developments. Generally speaking, more advanced patients have poorer prognosis. To integrate progression stage prediction systems with recurrence prediction systems, we proposed an ensemble prognostic model for colorectal cancer. In this model, each patient was assigned a most possible stage and a most possible recurrence status. If a patient was predicted to be recurrence patient in advanced stage, he would be classified into high risk group. The ensemble model considered both progression stages and recurrence status. High risk patients and low risk patients predicted by the ensemble model had a significant different disease free survival (log-rank test p-value, 0.0016) and disease specific survival (log-rank test p-value, 0.0041). The ensemble model can better distinguish the high risk and low risk patients than the stage prediction model and the recurrence prediction model alone. This method could be applied to the studies of other diseases and it could significantly improve the prediction performance by ensembling heterogeneous information.

## Introduction

Colorectal cancer (CRC) is one of the most malignant cancers. Its occurrence and progression involve complicated evolutionary process affected by multiple genes [1]. In America and Europe, CRC is the second most frequent cancer which leads to death ranking below lung cancer [2]. Early detection of CRC could reduce the morbidity and improve the prognosis [3].

CRC can be grouped into Dukes A, B, C, and D stages based on its developments [4]. Dukes' A carcinomas are those confined to the innermost lining of the colon or rectum with no invading into the extrarectal tissues and no metastases in lymph nodes. Dukes' B carcinomas are those that have invaded the musculature of the colon or rectum but have not yet involved the lymphatic system. Carcinomas of Dukes' C have spread to at least one regional lymph node. While carcinomas of Dukes' D have metastasized to somewhere else in the body such as the liver or lung [4]. In 1954, Dukes' stages B and C were further subdivided into B1, B2 and C1, C2 by the Americans Astler and Coller [5]. Type B1 carcinomas were those in which lesions have invaded into the muscularis propria with negative nodes, but without penetrating through. While type B2 carcinomas were those in which lesions have penetrated the muscularis propria with negative nodes. Carcinomas of type C1 have invaded into muscularis propria with positive nodes but have not penetrated through. While those of type C2 have penetrated through muscularis propria with positive nodes. Devised more than 70 years ago, the now modified Dukes' staging system provides adequate prognostic information for patients of stage A or D. However, the intermediate stages B and C are not so useful in discriminating good prognosis patients from poor ones [6].

So far, microarray analysis was used in several studies on primary tumor specimens to identify gene expression signatures predictive of CRC prognosis [6], [7]. The general approach for signature discovery was to analyze patients selected for good and poor outcomes, followed by assessment of the signature in additional cases. However, signature discovery based on outcome is generally confounded in patients undergoing adjuvant treatment. Therefore, it is difficult to distinguish markers of prognosis from those of therapy response [6], [7].

It has been reported that the expression differences between extreme stages of cancer could be used to predict recurrence in patients of intermediate stages. Advantages of this approach are that no follow-up data is required in tumor stage based discovery and that the confounding effect of previous therapy can be avoided by selecting patients who have not undergone adjuvant treatment [8].

In this study, by combining the progression stage prediction systems and the recurrence prediction systems, we proposed an ensemble prognostic model for CRC. High risk patients and low risk patients predicted by the ensemble model had a significant different disease free survival and disease specific survival. The performance of the ensemble model was better than that of the stage prediction model and recurrence prediction model alone.

## Materials and Methods

### Dataset

#### Gene expression profiles of different CRC stages

Gene expression profiles of 290 CRC patients were retrieved from NCBI Gene Expression Omnibus (GEO) (accession number: GSE14333), in which 44 patients belonged to Dukes stage A, 94 to stage B, 91 to stage C and 61 to stage D, respectively. The expression profiles were determined with Affymetrix Human Genome U133Plus 2.0 arrays interrogating 19621 genes.

#### Gene expression profiles of patients with and without recurrence

Gene expression profiles of 77 CRC patients with recurrence and 121 CRC patients without recurrence were retrieved from GEO (accession number: GSE12032). Patients with and without recurrence were denoted as 1 and 0, respectively. The expression profiles were determined with AceGene Human Oligo Chip 30K 1 Chip Version interrogating 10583 genes.

#### Gene expression profiles of patients with survival time

Gene expression profiles of 176 CRC patients with disease-free survival (DFS) and disease specific survival (DSS) were retrieved from GEO (accession number: GSE17538). The expression profiles were determined with Affymetrix Human Genome U133 Plus 2.0 Array interrogating 20068 genes.

These three datasets totally contained 644 CRC patients interrogating 10166 common genes. All the datasets were quantile normalized with “affy” package of R for further analysis [9].

### mRMR method

We used the Minimum Redundancy Maximal Relevance (mRMR) method to rank the importance of the features [10]. The mRMR method ranks features according both to their relevance to the target and the redundancy between features. A ranked feature with a smaller index indicates that it has a better trade-off between the maximum relevance to the target and minimum redundancy.

Both relevance and redundancy were quantified by mutual information (MI), which estimates the extent to which one vector is related to another. The MI is defined as:
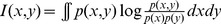
(1)where 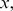 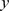
are vectors, 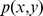
is their joint probabilistic density, and 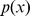
and 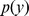
are the marginal probabilistic densities.

Suppose 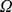 denotes the entire feature set, 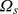 denotes the already-selected feature set containing *m* features and 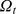 denotes the to-be-selected feature set containing *n* features. The relevance 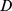 between a feature 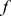 in 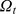 and the target 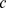 can be calculated by:
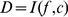
(2)


The redundancy
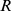
between the feature 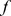
in 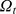 and all the features in 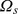 can be calculated by:
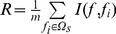
(3)


To determine the feature 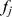 in 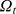
with the maximum relevance and minimum redundancy, the mRMR function combines equation (2) and equation (3) and is defined as:
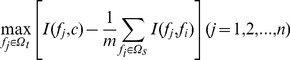
(4)


The mRMR feature evaluation will continue *N* rounds given a feature set with *N* (*N* = *m*+*n*) features. After the mRMR feature evaluation, a feature set
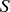
is obtained:
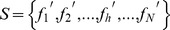
(5)where the index *h* of each feature indicates at which round the feature is selected. The smaller the index *h*, the earlier the feature satisfies equation (4) and the better the feature is.

### Nearest Neighbor Algorithm (NNA)

In this study, the Nearest Neighbor Algorithm (NNA), which has been widely used in bioinformatics and computational biology [3], [11], [12], [13], [14], was adopted to predict the class of colorectal tissue samples. We regarded each sample as a vector with the expression values of genes as its components. NNA calculates similarities between every two samples and makes its classification decision. In our study, the distance between two samples
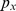
and
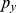
is defined as below:
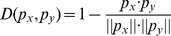
(6)where 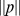 is the module of sample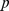. 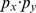 denotes the inner product of 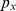 and 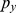. The smaller the 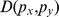 is, the more similar the two samples are.

In NNA, given a sample set
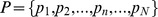
and a sample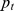, 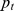 will be designated to the same class of its nearest neighbor 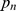 in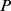, which is the sample having the smallest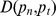:
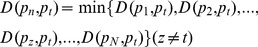
(7)


### Five-fold Cross-Validation Method

Five-fold cross-validation was often used to evaluate the performance of a classifier [15]. In five-fold cross-validation, all samples in the dataset are first divided equally into five parts. Subsequently, each part is in turn used for testing and the remaining 4-parts for training. Thus, each sample is tested exactly once.

To evaluate the performance of the predictor for CRC stages, the total accuracy (ACC) of prediction is calculated below:
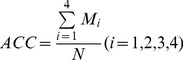
(8)where 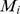 stands for the number of correctly classified patients for the *i*-th cancer stage, and 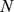 represents the total number of patients.

To evaluate the performance of the predictor for recurrence, the prediction accuracy, specificity, sensitivity and MCC (Matthews correlation coefficient) were calculated by:
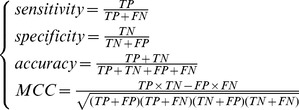
(9)where *TP*, *TN*, *FP*, *FN* denote the number of true positives, true negatives, false positives and false negatives, respectively.

### Incremental Feature Selection (IFS)

Based on the ranked features obtained by the mRMR method, we used the IFS [16], [17], [18], [19] approach to determine the optimal features. During the IFS procedure, features in the ranked feature list were added with a stepwise of 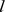 features from higher to lower rank. A new feature set was constructed when another 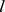 features were added. Totally 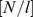 feature sets were composed from the *N* ranked features. The *i*-th feature set is:
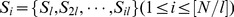
(10)where *N* denotes the total number of features in the original dataset and *l* is the number of features added in each step, which is a positive integer. In this study, we set 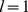. For each of the [*N*/*l*] feature sets, an NNA classifier was constructed and examined by using the five-fold cross-validation on the benchmark dataset. An IFS table was obtained with one column for the index *i* and the other columns for the prediction performance. The optimal feature set (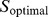) was selected, with which the predictor yielded the best prediction performance.

### Four different criteria for evaluating risk

In this study, 4 different criteria were proposed to assess the risk of CRC patients. In the first proposal, patients of stage B with recurrence as well as those of stage C and D irrespective recurrence status were considered high risk, while all other patients were regarded as low risk. In the second proposal, only cancer stages were considered. Patients of stage A were designated as low risk, while those of stage B, C and D as high risk. In the third proposal, patients of stage A and B were regarded as low risk, while those of stage C and D were high risk. In the last proposal, only recurrence information was utilized. Patients with recurrence were regarded as high risk, while patients without recurrence were considered low risk. Subsequently, Kaplan–Meier estimator [20] was employed to evaluate the significance of disease free survival and disease specific survival between high and low risk groups.

## Results

### Model for predicting CRC stages

After running the mRMR software, we obtained two tables (see File S1). One was called MaxRel table ranking the genes according to their relevance to the stages of samples. The other was called mRMR feature table listing the genes with the maximum relevance and minimum redundancy to the stages of samples.

**Figure 1:**
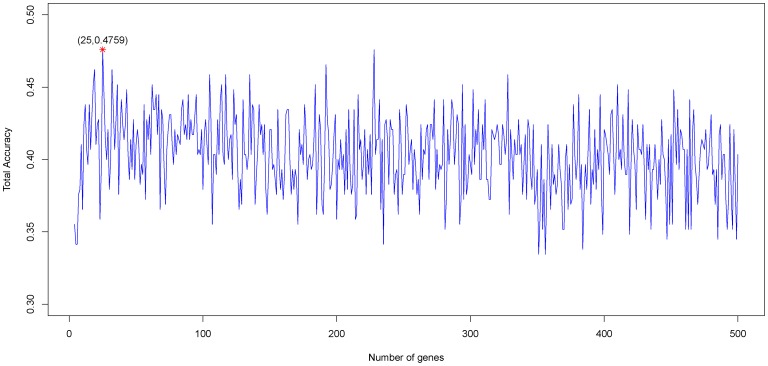
IFS curve showing the overall prediction accuracies versus gene numbers for CRC stages prediction model. The IFS curves were drawn based on the data in File S2. The overall prediction accuracy reached the peak when the number of genes was 25. The 25 genes thus obtained were used to compose the optimal gene set for the CRC stage predictor.

On the basis of the outputs of mRMR, we constructed 500 feature subsets according to Eq.10. An NNA predictor for each subset was modeled correspondingly, which was then evaluated by five-fold cross-validation. The number of features used in each predictor was 1,2,3,…, as described in Materials and Methods section. The IFS result can be found in Supporting Information S2. Shown in Fig. 1 is the IFS curve plotted based on Supporting Information S2. The x-axis is the number of genes used for classification, and the y-axis is the prediction accuracies of the nearest neighbor predictors evaluated by five-fold cross-validation. The maximum accuracy reached 0.4759 when 25 genes were utilized. These 25 genes were regarded as the optimal biomarkers for the prediction of CRC stages (see the top 25 genes in the “mRMR Table” of File S1).

### Model for predicting CRC recurrence

**Figure 2:**
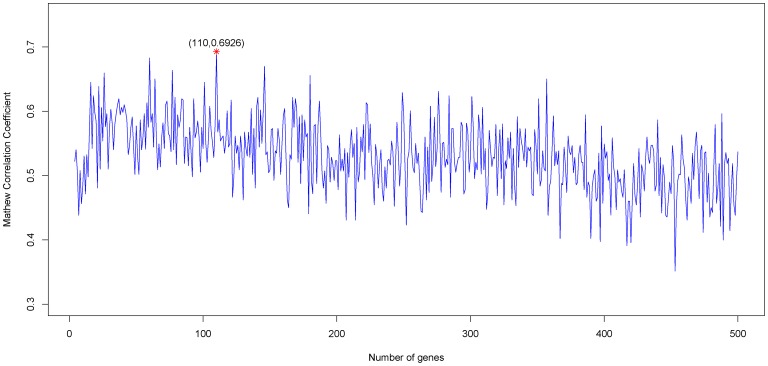
IFS curve showing the MCC versus gene numbers for CRC recurrence prediction model. The IFS curves were drawn based on the data in File S4. The MCC reached the peak when the number of genes was 110. The 110 genes thus obtained were used to compose the optimal gene set for the CRC recurrence predictor.

Similarly, by using the mRMR method, we also obtained MaxRel and mRMR tables for CRC recurrence prediction (see File S3). Based on these two tables, 500 feature subsets were constructed according to Eq.10. An NNA predictor was modeled for each subset and was evaluated by five-fold cross-validation. Shown in Fig. 2 is the IFS curve plotted based on the data in Supporting Information S4. The x-axis is the number of genes used for the classification, and the y-axis is the MCC values of classifiers evaluated by five-fold cross-validation. The maximum MCC was 0.6926 when 110 genes were utilized. With such a classifier, the prediction sensitivity, specificity and accuracy were 0.8182, 0.8760 and 0.8535, respectively. These 110 genes were regarded as the optimal biomarkers for the prediction of CRC recurrence (see the top 110 genes in the “mRMR Table” of File S3).

### Comparing survival time of four different criteria for evaluating risk

We used the two models mentioned above to predict cancer stages and recurrence status of patients with survival times. These patients were divided into high risk and low risk groups according to the four different criteria. The survival times of patients for the four proposals were compared.

**Figure 3:**
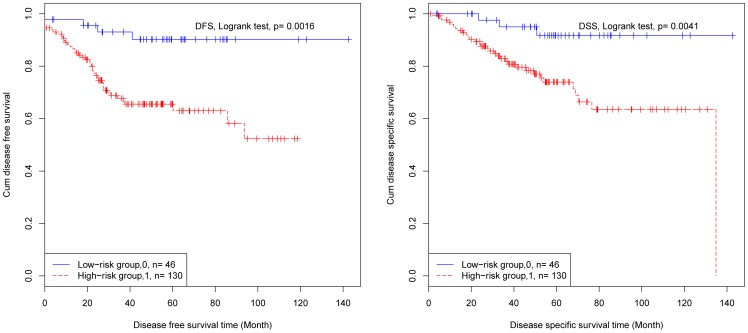
Survival curve for the first proposal. (A) Disease free survival (log-rank test, p-value = 0.0016). (B) Disease specific survival (log-rank test, p-value = 0.0041).

According to the first proposal, there was a significant difference in disease-free survival (DFS) time (logrank test, p-value = 0.0016) and disease specific survival (DSS) time (logrank test, p-value = 0.0041) between high risk and low risk groups (Table 1 and Figure 3). For the second proposal, there is also a significant difference in DFS (logrank test, p-value = 0.0021) and DSS (logrank test, p-value = 0.0055) between high and low risk groups. However, it is not as significant as that of the first proposal (Table 1). For the third proposal, the difference in DFS (logrank test, p-value = 0.3313) and DSS (logrank test, p-value = 0.2556) was not significant between high and low risk groups (Table 1). According to the last proposal, the difference in DFS (logrank test, p-value = 0.5525) and DSS (logrank test, p-value = 0.5606) between high and low risk patients was not significant, either (Table 1).

**Table 1:** Survival time comparison of four different criteria for evaluating risk.

	DFS logrank p-value	DSS logrank p-value
Proposal 1	0.0016	0.0041
Proposal 2	0.0021	0.0055
Proposal 3	0.3313	0.2556
Proposal 4	0.5525	0.5606

DFS: disease-free survival. DSS: disease specific survival.

## Discussion

### 25 CRC stage related candidate genes

In this study, we identified 25 candidate genes that can be used to distinguish CRC patients from different stages (see the top 25 genes in the “mRMR Table” of File S1). Some of the genes have already been reported to be related to CRC. Below, we will briefly discuss their relationships with CRC.

ZC3H12A (zinc finger CCCH-type containing 12A), is an MCP1 (CCL2; MIM 158105)-induced protein that acts as a transcriptional activator and causes cell death of cardiomyocytes, possibly via induction of genes associated with apoptosis. Recently, ZC3H12A has been identified as one of the upregulated genes in S100A8/A9-activated colon tumor cells, whose products promote leukocyte recruitment, angiogenesis, tumor migration, wound healing, and formation of premetastatic niches in distal metastatic organs [21].

CADPS encodes a novel neural/endocrine-specific cytosolic and peripheral membrane protein required for the Ca^2+^-regulated exocytosis of secretory vesicles. Recently, CADPS has been detected to harbor mutation p.R722W and p.R787* in colon carcinoma [22]. Furthermore, this gene has been reported increased expression in ovary compared to omentum in 47 samples analyzed by microarray [23].

FZD8 (Frizzled-8) is a member of the frizzled gene family, which are transmembrane receptors transducing Wnt signals based on ligand-dependent preferentiality for caveolin- or clathrin-mediated endocytosis [24], [25]. The Wnt signaling pathway has been well documented to be related to CRC [26], [27], [28]. In addition, recently FZD8 has been proposed to be a putative therapeutic target in human lung cancer [29]. Furthermore, it has been shown that Frizzled-10, a member of rizzled gene family, is up-regulated in primary CRC, and is a positive regulator of the WNT-beta-catenin-TCF signaling pathway [30].

CAV3 (caveolin 3) as well as Caveolin-1, -2 are the principal proteins of caveolae, the vesicular invaginations of the plasma membrane. Studies have suggested that caveolins played an important role in cellular signaling and, possibly, in tumorigenesis [31]. Recently, Caveolin-1, as a member of caveolin family, has been reported to act as an anti-apoptotic protein in colon cancer cells by binding to Ku70 and inhibiting Bax-dependent cell death [32]. In addition, it has been shown that loss of Caveolin-3 can induce a lactogenic microenvironment that is protective against mammary tumor formation [33].

PACS2 (phosphofurin acidic cluster sorting protein 2) has been reported to be required for efficient apoptosis of colorectal cell lines. PACS-2 is an essential TRAIL effector, required for killing colon cancer cells in vitro and virally infected hepatocytes in vivo [34], [35].

SOX11 encodes a member of the SRY-related HMG-box family of transcription factors involved in the regulation of embryonic development and in the determination of the cell fate. Expression of Sox4 and Sox11 has been shown to increase in many different types of human cancers, including basal cell carcinomas (BCC) and medulloblastomas. The carcinomas can attack various places in human body, including liver, prostate, ovary, lung, and colon. In addition, highly specific overexpression of the transcription factor SOX11 has been detected in human malignant gliomas [36]. Furthermore, SOX11 expression correlates to promoter methylation and regulates tumor growth in hematopoietic malignancies [37].

PRKCDBP encodes a binding protein of the protein kinase C, delta (PRKCD). PRKCDBP is a proapoptotic tumor suppressor which is commonly altered in CRC by promoter hypermethylation, and its gene transcription is directly activated by NF-κB in response to TNFα, which suggests that PRKCDBP inactivation may contribute to tumor progression by reducing cellular sensitivity to TNFα and other stresses, particularly under chronic inflammatory microenvironment [38].

### 110 CRC recurrence related candidate genes

In this study, we identified 110 candidate genes that can be used to predict CRC recurrence (see the top 110 genes in the “mRMR Table” of File S3). Some of the genes have been reported to be related to CRC. Below, we will briefly discuss their relationships with CRC.

SLC26A2 encodes a protein belonging to the solute carrier family. The diastrophic dysplasia sulfate transporter is a transmembrane glycoprotein implicated in the pathogenesis of several human chondrodysplasias. SLC26A2 has been detected to be downregulated in colon cancer biopsies compared with surrounding normal tissue [39]. In addition, SLC26A2 and SLC6A14 mRNA levels have been used as part of a seven gene panel yielding rates of correct prediction, sensitivity, and specificity higher than that with previously available diagnostic indices for ulcerative colitis, Crohn's disease, and irritable bowel syndrome [40].

SLPI (secretory leukocyte peptidase inhibitor) encodes a secreted inhibitor which protects epithelial tissues from serine proteases. Higher SPLI transcript expression has been detected in many cancer cell lines, including ovarian, renal and colon carcinoma by using RTQ-PCR expression analysis [41]. In addition, a hypothesis has been proposed that a fully human monoclonal antibody that neutralizes SLPI's protease activity may be therapeutically useful for ovarian and colon carcinoma [41].

CLEC4M encodes a transmembrane receptor and is often referred to as L-SIGN because of its expression in the endothelial cells of the lymph nodes and liver. CLEC4M has been identified as one of the genes that differentially expressed in patient colon cancer hepatic metastasis specimens and its xenograft [42]. In addition, CLEC4M significantly differentially expressed in malignant versus non-malignant breast tissue [43].

ISCU encodes the two isomeric forms, ISCU1 and ISCU2, of the Fe-S cluster scaffold protein, which are necessary for several mitochondrial enzymes and other subcellular compartment proteins. It has been shown that extra-mitochondrial localisation of frataxin and its association with ISCU1 play a key role in enterocyte-like differentiation of the human colon adenocarcinoma cell line Caco-2 [44]. In addition, by targeting ISCU, miR-210 decreases the activity of Kreb's cycle enzymes and mitochondrial function, contributes to an increase in free radical generation in hypoxia, increases cell survival under hypoxia, induces a switch to glycolysis in both normoxia and hypoxia, and upregulates the iron uptake required for cell growth. Notably, analysis of more than 900 patients with different tumor types, including breast cancer, showed that the suppression of ISCU was correlated with a worse prognosis [45].

PANK2 encodes a protein belonging to the pantothenate kinase family and is the only member of that family to be expressed in mitochondria. It has been reported that the loss of one subset of kinases including PANK2 resulted in reduced β-cat–dependent transcription in colon carcinoma cells, representing potential targetable therapeutic genes [46].

### Protein-protein interaction between stage related genes and recurrence related genes

**Figure 4:**
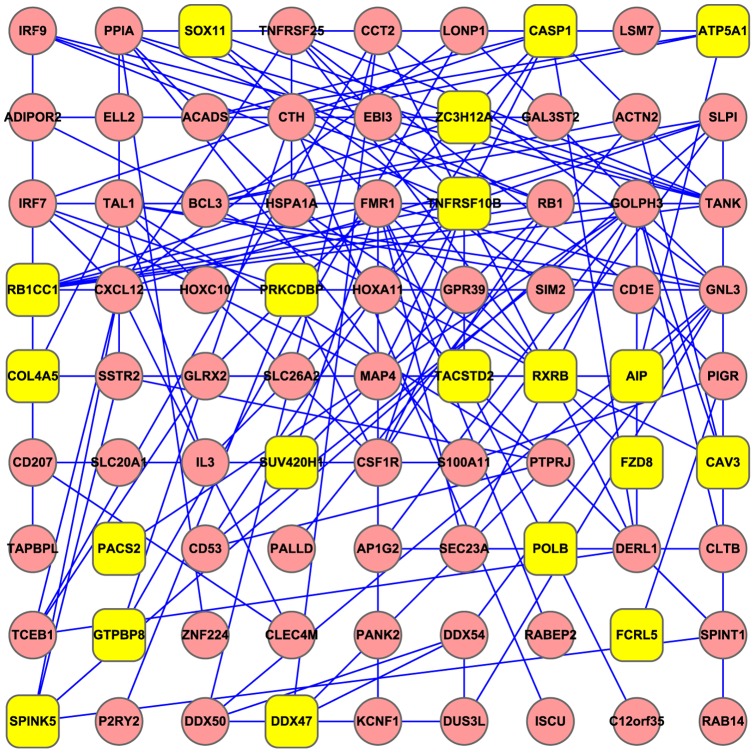
Protein-protein interaction network between stage related genes and recurrence related genes. Yellow round rectangles represent stage related genes while red elipses represent recurrence related genes. 20 of the 25 stage related genes and 61 of the 110 recurrence related genes were presented.

We mapped the 25 stage related genes and the 110 recurrence related genes to protein-protein interaction network constructed based on data from STRING [47]. From Fig. 4, it can be seen that close connections existed between stage and recurrence related genes. It has been shown that the stage related gene RB1CC1 (RB1-inducible coiled-coil 1) can suppress cell cycle progression and inhibit proliferation through activating the promoter and expression of recurrence related gene RB1 in human cancer [48], [49], [50]. In addition, the interaction between these two genes was also involved in the prognosis of cancer patients. It has been reported that RB1CC1 together with RB1 and p53 play important roles in long-term survival of Japanese breast cancer patients [51]. Besides, Paun et al. have revealed RB1CC1 as a novel target of frequent mutation and aberrant upregulation in MSI-H (high level of microsatellite instability) CRC [52]. Therefore, it is plausible to assume that the interaction between these two genes is also implicated in the tumorigenesis and the prognosis of CRC. Moreover, the interaction between stage related gene ZC3H12A and recurrence related gene TANK was also supported by a large scale of protein-protein interaction study [53]. Overall, the interactions between these candidate genes may account for the etiology of CRC and influence the prognosis.

### Prospect of our method

In the former prognosis models, no recurrence was considered to be equivalent to low risk. However, from our point of view, the prognosis of the advanced CRC patients of stage C or D could be worse even though they were predicted to not recur. On the contrary, patients from stage A may survive anyway though they were predicted to recur. Therefore, in our study, we proposed a novel criterion to evaluate the risk level, in which patients predicted to recur in stage B as well as patients of stage C and D irrespective recurrence status were considered high risk. All other patients were regarded as low risk. It was revealed that the survival time between high and low risk patients assessed by this criterion was more significant than that derived by only considering recurrence status or tumor stages.

It can be seen that when only considering tumor stages, the difference of survival time in the second proposal was significant but it was not the case in the third one. The reason could be that accurate classification of patients in intermediate-stages remains problematic. Eschrich et al. have shown that survival curves grouped by both Dukes' stage B and C can be further subdivided into good and poor prognosis groups (ref Fig. 2) [6]. That is why the survival time difference was significant when only patients of stage A were designated as low risk.

Overall, the first proposal outperformed the other three because it integrated cancer stages and recurrence status, rather than considering only one of them. This method could be applied to the studies of other diseases and it could significantly improve the prediction performance by ensembling heterogeneous information.

## Supporting Information

File S1 **mRMR result for CRC stage prediction model.** This file contains two sheets. The first one is the MaxRel feature table, which ranked the top 500 genes according to the relevance between features and class of the samples. The second one is the mRMR feature table, which ranked these 500 genes according to the redundancy and relevance criteria.
(XLSX)

File S2 **The prediction accuracy for each tumor stage and overall prediction accuracy for all tumor stages at each run of IFS.**
(XLSX)

File S3 **mRMR result for CRC recurrence prediction model.** This file contains two sheets. The first one is the MaxRel feature table, which ranked the top 500 genes according to the relevance between features and class of the samples. The second one is the mRMR feature table, which ranked these 500 genes according to the redundancy and relevance criteria.
(XLSX)

File S4 **The sensitivity (Sn), specificity (Sp), accuracy (Ac), Matthews correlation coefficient (MCC) of each run of IFS for CRC recurrence prediction model.**
(XLSX)
